# Supplementary material for: Empirical methods for controlling false positives and estimating confidence in ChIP-Seq peaks
Source: BMC Bioinformatics. 2008 Dec 5;9:523. doi: 10.1186/1471-2105-9-523 (PMC2628906; doi:10.1186/1471-2105-9-523)
Supplement: Additional File 1 — A variety of html documents from the USeq web site detailing the available applications, their best usage, output file type descriptions, command line menus, etc. [file 1471-2105-9-523-S1.zip › USeqUserGuides/JavaDoc.zip!/JavaDoc/util/gen/MD5.html]

MD5


|  |  |  |
| --- | --- | --- |
| **Overview** \| **Package** \| **Class** \| **Use** \| **Tree** \| **Deprecated** \| **Index** \| **Help** | |  |
| **PREV CLASS**   **NEXT CLASS** | **FRAMES**    **NO FRAMES**     **All Classes** |  |
| SUMMARY: NESTED \| FIELD \| CONSTR \| METHOD | DETAIL: FIELD \| CONSTR \| METHOD |  |


---


## util.gen Class MD5

```
java.lang.Object
  java.security.MessageDigestSpi
      java.security.MessageDigest
          util.gen.MD5
```

**All Implemented Interfaces:**: java.lang.Cloneable

---

``` public class MD5 extends java.security.MessageDigest implements java.lang.Cloneable ```

This class implements the MD5 algorithm as described in RFC1321.

**See Also:**: `MessageDigest`

---

**Constructor Summary**

`MD5()`


| **Method Summary** | |
| --- | --- |
| `java.lang.Object` | `clone()` |
| `byte[]` | `engineDigest()` |
| `int` | `engineGetDigestLength()` |
| `void` | `engineReset()` |
| `void` | `engineUpdate(byte b)` |
| `void` | `engineUpdate(byte[] bytes, int off, int len)` |

**Methods inherited from class java.security.MessageDigest**

`digest, digest, digest, getAlgorithm, getDigestLength, getInstance, getInstance, getInstance, getProvider, isEqual, reset, toString, update, update, update, update`

**Methods inherited from class java.lang.Object**

`equals, getClass, hashCode, notify, notifyAll, wait, wait, wait`

**Constructor Detail**

### MD5

```
public MD5()
```


**Method Detail**

### clone

```
public java.lang.Object clone()
```

:   **Overrides:**: `clone` in class `java.security.MessageDigest`

---


### engineGetDigestLength

```
public int engineGetDigestLength()
```

:   **Overrides:**: `engineGetDigestLength` in class `java.security.MessageDigestSpi`

---


### engineReset

```
public void engineReset()
```

:   **Specified by:**: `engineReset` in class `java.security.MessageDigestSpi`

---


### engineUpdate

```
public void engineUpdate(byte b)
```

:   **Specified by:**: `engineUpdate` in class `java.security.MessageDigestSpi`

---


### engineUpdate

```
public void engineUpdate(byte[] bytes,
                         int off,
                         int len)
```

:   **Specified by:**: `engineUpdate` in class `java.security.MessageDigestSpi`

---


### engineDigest

```
public byte[] engineDigest()
```

:   **Specified by:**: `engineDigest` in class `java.security.MessageDigestSpi`


---


|  |  |  |
| --- | --- | --- |
| **Overview** \| **Package** \| **Class** \| **Use** \| **Tree** \| **Deprecated** \| **Index** \| **Help** | |  |
| **PREV CLASS**   **NEXT CLASS** | **FRAMES**    **NO FRAMES**     **All Classes** |  |
| SUMMARY: NESTED \| FIELD \| CONSTR \| METHOD | DETAIL: FIELD \| CONSTR \| METHOD |  |


---
